# Transcranial direct current stimulation combined with a brief intervention for smoking cessation: a randomized double-blind clinical trial

**DOI:** 10.1007/s00406-023-01705-8

**Published:** 2023-11-13

**Authors:** Ulrich Palm, Mark Obergfell, Andrea Rabenstein, Jonas Björklund, Gabi Koller, Frank Padberg, Tobias Rüther

**Affiliations:** 1 Department of Psychiatry and Psychotherapy, LMU University Hospital Munich, Nußbaumstraße 7, 80336 Munich, Germany; 2 Medical Park Chiemseeblick, Bernau-Felden, Germany

**Keywords:** tDCS, Brain stimulation, Tobacco, Nicotine dependence

## Abstract

Non-invasive brain stimulation methods are currently being evaluated for treatment of addictive disorders. Some evidence indicates that modulating left and right prefrontal brain activity by transcranial direct current stimulation (tDCS) can reduce craving and relapse rates in tobacco addiction. Therefore, this study investigated the effects of active and sham tDCS as an add-on treatment to a standardized brief intervention for smoking cessation. This randomized, double-blind study included 36 participants (22 women and 14 men) with nicotine dependence according to ICD-10 criteria. At five visits on alternate days, participants underwent a 20-min active or sham tDCS over the left dorsolateral prefrontal cortex and subsequently participated in a 10-min brief intervention for smoking cessation. Patients were followed up after 3 months. On each treatment day and at follow-up, abstinence was assessed as the smoking status *nonsmoker* and craving was assessed with the German version of the Questionnaire on Smoking Urges. At each visit, the number of cigarettes smoked per day was recorded and carbon monoxide in expired air and cotinine in saliva were measured. At follow-up, a study-specific questionnaire was used to assess tobacco use. All 36 participants completed the treatment sessions, but one participant in each group was lost to follow-up. Abstinence rates were not significantly different between the groups at any of the study visits, but craving was significantly lower in the active group at tDCS session 5 compared with session 1. tDCS combined with a brief intervention may support smoking cessation, but studies need to evaluate whether longer and more intensive treatment can achieve significant, sustainable effects.

## Introduction

Tobacco use is an important risk factor for cardiovascular diseases; respiratory diseases such as lung cancer and chronic obstructive pulmonary disease; stroke; and damage to the periodontium [1]. Although it is an avoidable risk, in 2019 it resulted in about 7.69 million deaths globally [2]. The established treatments for tobacco dependence include standardized psychotherapeutic interventions and administration of nicotine replacement products. However, new treatment approaches are urgently needed because of the high relapse rates.

Imaging studies have shown that stimulus-induced craving for smoking is associated with changes in the DLPFC [3]. Craving plays a key role in achieving and maintaining abstinence because it is the main reason why many people start smoking again [4, 5]. For example, an analysis of 2600 smokers showed that abstinence or relapse depended mainly on the amount of craving for smoking [5]. In a study in 64 former smokers, Swan et al. [6] found a significant association between abstinence and relapse. The same study also showed that the amount of craving is associated with the time until relapse.

One potential treatment method is transcranial direct current stimulation (tDCS), a noninvasive brain stimulation procedure that applies a direct current to the scalp through electrodes to modulate the neural activity of the underlying brain regions and their connected areas [7, 8]. The respective polarity (anode or cathode) determines the effects of the stimulation: Anodal stimulation increases the neuronal excitability of the cells, whereas cathodal stimulation decreases it [8–11]. In both cases, tDCS changes the excitability of neurons by affecting neuroplasticity, similar to long-term potentiation (LTP) and long-term depression (LTD), whereby the changes depend on the duration and intensity of the stimulation [8, 12]. Because effects are non-linear, increasing the duration of anodal tDCS can also decrease neuronal excitability [8, 13] and increasing the strength of the electric current used for cathodal stimulation can increase excitability [8, 14].

The ability of tDCS to change the membrane potential of neurons and induce action potentials in cells has been investigated and proven in numerous pre-clinical and clinical studies [8, 15–17]. In addition, tDCS can regulate cell migration of various types of cells and influence the direction of growth and differentiation of cells [18, 19]. The mechanisms of action and effects of tDCS in the different types of cells are largely unknown; however, effects on cell alignment, cell migration speed, and neurite growth have been shown, and research indicates that they may be caused by changes in intracellular Ca^2+^ concentrations [20, 21].

Since the 1960s, research has evaluated the effect of tDCS on various brain regions. Studies were performed not only in animals but also in healthy volunteers and patients with tobacco, alcohol, and marijuana dependence (tobacco [3, 22], alcohol [23], marihuana [24]). For example, in a randomized placebo-controlled double-blind study, Fregni et al. [3] applied anodal stimulation with active or sham tDCS to 24 smokers to stimulate the left and right dorsolateral prefrontal cortex (DLPFC). The researchers assessed whether tDCS affected the level of craving by showing participants a smoking video. Compared with baseline, the study found a significant reduction in smoking craving after active tDCS of the left and right DLPFC, whereby the reduction was greater with than without stimulus-induced craving. In a randomized placebo-controlled clinical study in 27 individuals, Boggio et al. [22] found a significant reduction of craving for smoking in the active tDCS group compared with the sham tDCS group. All 27 participants received anodal tDCS of the left DLPFC for 5 days. During the 5-day tDCS stimulation, the verum group showed a small but significant decrease in the number of cigarettes smoked compared with the placebo group.

In another randomized placebo-controlled double-blind study, Boggio et al. [23] applied anodal and cathodal active or sham tDCS to the DLPFC of 13 individuals with alcohol dependence. Stimulation was applied for 30 s, and participants were shown videos about alcohol use. The study showed a significant reduction of alcohol craving with both anodal and cathodal stimulation of the DLPFC in the verum group compared with the placebo group. In another placebo-controlled study by Boggio et al. [24], anodal and cathodal active or sham tDCS of the DLPFC was applied in 25 people with chronic marihuana dependence, and a significant reduction in marijuana craving was shown in the verum group.

Therefore, it may be postulated that tDCS treatment can indirectly affect behavior and thus also increase the abstinence rate by reducing craving for smoking. On the basis of the findings that tDCS affects neuronal membrane potentials and modulates the excitability of nerve cells, including in the DLPFC, we hypothesized that providing targeted information to smokers in the form of a brief intervention on smoking cessation directly after tDCS may increase the abstinence rate and reduce smoking craving to a similar or even larger degree than was shown for tDCS alone. In addition, it may increase not only the treatment efficiency but also patients’ motivation for and willingness to participate in smoking cessation interventions. In treatment settings, successful smoking cessation programs aim to achieve not only complete abstinence but also a reduction in smoking; consequently, clinical studies on smoking need to assess both the cessation rate and craving for smoking.

Thus, we performed a clinical study to test the hypothesis that smokers treated by active tDCS and a brief intervention on smoking cessation have significantly less craving for smoking and significantly higher abstinence rates than smokers treated by sham tDCS and the intervention alone.

## Methods

### Study design

This randomized, placebo-controlled, double-blind study was performed at the Department of Psychiatry and Psychotherapy of the Ludwig Maximilian University Munich. Participants were randomized 1:1 to 5 sessions of sham or active tDCS and were followed up after 3 months.

The study was approved by the ethics committee of the Ludwig Maximilian University Munich (approval number 513-13) and was performed in accordance with the International Council for Harmonisation Good Clinical Practice guidelines and the principles of the Declaration of Helsinki.

### Participants

A total of 36 individuals participated in the study (22 women and 14 men). The sample size was determined in accordance with the recommendation by Lancaster et al. [25] to include at least 30 individuals in a clinical study. Participants were recruited by newspaper advertisements in a local weekly newspaper and by information on the homepage of the specialized outpatient clinic for tobacco dependence at the Ludwig Maximilian University Munich.

The inclusion criteria were as follows: nicotine dependence according to ICD-10 criteria (F17.2); smoker for at least 1 year; 10 or more cigarettes/day; a nicotine dependence score greater than 4 on the Fagerström Test (FTND) [26, 27]; a carbon monoxide (CO) value greater than 10 ppm (measured in expired air by a Micro + Smokerlyzer [Bedfont Scientific Ltd.]); older than 18 years old; provided written informed consent; and no attempt at smoking cessation or drug treatment for smoking cessation for at least 3 months before the start of the study. Exclusion criteria were the clinical diagnosis of an acute mental disorder according to ICD-10/DSM-IV; having a legal representative; pregnancy; chronic mental disorder; acute risk of suicide; drug, medication, or alcohol abuse at the time of the study; dementia according to ICD-10/DSM-IV criteria (clinical diagnosis) [28]; history of severe traumatic brain injury; evidence of structural damage to the basal ganglia or brain stem; severe neurological diseases (such as prolapsed disc in the past 6 months; polyneuropathies; Parkinson syndrome; epilepsy; systemic neurological diseases; cerebrovascular diseases; history of stroke; gradually worsening, repeated cerebral ischemia; increased intracranial pressure; normal pressure hydrocephalus); severe medical diseases (such as manifest arterial hypertension, severe heart disease, pacemaker, respiratory insufficiency); any type of electronic implant; any type of malignancy, either previous or current; severe active infection; chronic and systematic dermatological diseases; and bone diseases (such as Paget disease, osteoporosis with spontaneous fractures, new fractures).

All participants provided written informed consent to participate in the study. Participants who completed the study received a one-time financial compensation of 100 euros.

### Randomization

Participants were assigned to active or sham tDCS in a 1:1 ratio by an independent investigator (UP) with a double-blind randomization procedure, Software RandList (Version 1.2, DatInf GmbH, Tübingen, Germany). This computer program uses a random number generator to create a list of 4-digit numbers and assigns each participants a number (pseudonym) from this list. All study participants and study investigators were blind to the group.

### Treatments

#### tDCS stimulation

Participants received 5 separate tDCS stimulations with a CE-certified Eldith DC Stimulator (neuroConn GmbH, Ilmenau, Germany). The anode was attached over the left DLPFC in a position corresponding to the left electroencephalogram (EEG) F3 location (according to the 10–20 system), and the cathode was attached over the right supraorbital cortex in a position corresponding to the EEG Fp2 location. Stimulation was performed with a 2 mA current for 20 min, with additional ramp-in and ramp-out phases of 15 s each. The ramp-in and ramp-out phases were identical in active and sham tDCS. In sham tDCS, the stimulator was pre-programmed to generate a 20-min *off* interval between the ramp-in and ramp-out phases; the sensation of the *off* interval is identical to the sensation of active stimulation [29].

All persons performing the stimulation were trained in the use of the tDCS stimulator and blind to the type of stimulation. Active or sham tDCS was activated by entering the participant’s number from the randomization list into the apparatus.

For each participant, tDCS sessions were performed at 5 visits every other day over a 9-day period (V1, V3, V5, V7, and V9). Five sessions were considered to be sufficient because Boggio et al. [22] showed in a clinical study that 5 days of anodal active tDCS over the left DLPFC resulted in a significant reduction of smoking craving and a significant decrease in the number of cigarettes smoked, and the 5 sessions were performed every other day for organizational reasons.

#### Brief intervention

Immediately after each of the 5 tDCS treatments, each participant attended a 10-min brief intervention for smoking cessation. To standardize the quality of the brief intervention, all interventions were performed by the same investigator. The content of the brief intervention was based on the brochure “The Smoke-free Program” (authors’ translation) and the accompanying instructor manual “Compact version. The Smoke-free Program,” and participants received also a written copy of the brochure [30, 31]. The topics discussed with participants at each brief intervention are shown in Table 1.

**Table 1:** Topics discussed with participants during the brief interventions for smoking cessation at study visits 1 to 5

Visit	Brief intervention topics			
Visit 1	Preparing to stop smoking	List to prepare for my first smoke-free 24 h	Advantages and disadvantages of smoking	My goals
Visit 2	Timetable for my first smoke-free 24 h	On the day after stopping smoking		
Visit 3	What to expect after stopping smoking	Decatastrophizing	Building up motivation	
Visit 4	My alternatives	Alternatives for my hands, mouth, head, and body	Smoking and body weight	
Visit 5	Relapses and incidents	Handing over of an emergency card	How to behave if something happens	

### Clinical assessments and ratings

Before each tDCS stimulation (V1, V3, V5, V7, and V9), the CO content of expired air was measured with a Mikro-Smokelyzer (Bedfont Scientific Ltd.). In addition, a sterile salivette device (Salivette, Sarstedt AG & Co., Nümbrecht, Germany) was used to obtain a saliva sample for a cotinine test: Participants were instructed to place a cotton roll in their mouths, to chew on it for 1 min, and then to spit it into a tube without touching it. The salivettes were kept frozen in the laboratory until analysis.

After every stimulation, participants were asked the questions in the German version of the Questionnaire on Smoking Urges (QSU) [32]. The QSU comprises 32 items that are rated on a scale ranging from 1 (strongly disagree) to 7 (strongly agree). The items are assigned to either the Factor 1 scale, which assesses the “desire and intention to smoke and the anticipation of a positive outcome of smoking” [32, 33], or the Factor 2 scale, which “describes the anticipation of immediate relief from nicotine withdrawal or relief from negative effect and the strong urge to smoke” [32]. In addition, participants completed the self-rated Comfort Rating Questionnaire (CRQ) to assess side affects of the stimulations [34].

At the 90-day follow-up (V6), 7-day and continuous abstinence were assessed with a study-specific questionnaire comprising 5 questions on tobacco use (see Appendix). In addition, abstinence was validated with a CO test, a saliva cotinine test was performed, the QSU was completed, and the number of cigarettes smoked per day was recorded.

The parameters assessed at each visit are summarized in Table 2.

**Table 2:** Parameters recorded at each study visit

	Day 1 (V1)	Day 3 (V2)	Day 5 (V3)	Day 7 (V4)	Day 9 (V5)	Day 90 (V6)
Screening of inclusion and exclusion criteria, patient information, informed consent, randomization	X					
FTND	X					
Cigarettes/day	X	X	X	X	X	X
CO measurement	X	X	X	X	X	X
Cotinine test	X	X	X	X	X	X
tDCS	X	X	X	X	X	
Brief intervention	X	X	X	X	X	
QSU	X	X	X	X	X	X
CRQ	X	X	X	X	X	
Assessment of tobacco use						X

CO, concentration of carbon monoxide (ppm) in expired air; CRQ, Comfort Rating Questionnaire; FTND, Fagerström Test of Nicotine Dependence; QSU, Questionnaire on Smoking Urges; tDCS, transcranial direct current stimulation

#### Primary endpoints

Primary endpoints were the abstinence rate, i.e., the smoking status *nonsmoker*, and craving. Abstinence rate was assessed as the number of participants who were nonsmokers after each of the 5 intervention sessions and at the 3-month follow-up. The abstinence rate and smoking status *nonsmoker* were assessed at each of the 5 tDCS visits by recording the number of cigarettes smoked per day, the cotinine level in saliva, and the CO level in expired air. At follow-up, they were assessed by recording the continuous and 7-day abstinence rate with the questionnaire on tobacco use. Participants were classified as a *nonsmokers* if they smoked zero cigarettes a day, had a CO level less than or equal to 5, and showed a marked decrease in the cotinine level.

Craving for tobacco was assessed with the German version of the QSU at each of the 5 study visits and at follow-up.

#### Secondary endpoint

The secondary endpoint was the number of cigarettes smoked per day, which was recorded at each of the 6 study visits by asking the participants.

### Statistical analysis

Data were analyzed with SPSS version 22.0 (SPSS Inc., U.S.A.). A per protocol analysis was performed. Because the overall sample size was less than 50 (*N* = 36), the Shapiro–Wilk test was applied to test for normal distribution. Because of the small sample size, group differences in nominal data (sex, smoking status) were analyzed with the Fisher test, and group differences in metric data, with the Mann–Whitney *U* test. To analyze the interaction effects over the course of study visits 1–6, the active and sham values at the individual visits were analyzed separately by Friedman’s two-factor analysis of variance in related samples. A *P* value of <0.05 was considered as statistically significant. Bonferroni correction for multiple testing was considered for final analysis but omitted as all results were insignificant.

## Results

### Participant flow and losses

**Fig. 1:**
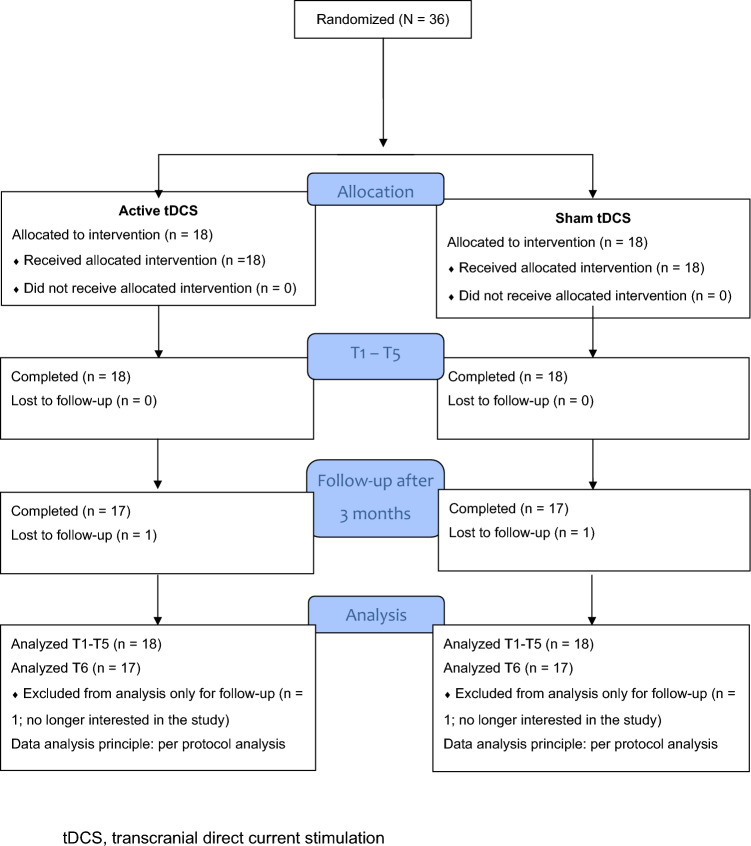
CONSORT flowchart of the study. tDCS, transcranial direct current stimulation

All 36 participants successfully completed the 5 tDCS and brief consultation sessions. Only 1 participant in the active group and 1 in the sham group was lost to follow-up because they were no longer interested in the study and refused to participate. Figure 1 shows the CONSORT flowchart of the study [35].

### Demographic and baseline data

The demographic and baseline data are shown in Table 3. Both groups included more women than men, but the difference was not statistically significant. Furthermore, the FTND score and cotinine level were higher in the sham group than in the active group, and the CO level was higher in the active group; again, none of the differences was significant.

**Table 3:** Demographic and clinical data at baseline visit

	Active tDCS	Sham tDCS	Statistical test and *p* value
N	18	18	
Age, mean (SD), years	51.39 (12.28)	50.56 (15.5)	T_(34)_ = −0.179; *p* = 0.859
Sex			
Female, n (%)	12 (66.7%)	10 (55.6%)	Fisher’s exact test, *p* = 0.733
Male, n (%)	6 (33.3%)	8 (44.4%)	
FTND sum score, mean (SD)	4.72 (2.40)	5.50 (2.33)	U = 121.500; *p* = 0.194
Cigarettes per day, mean (SD), n	21.44 (11.62)	21.61 (7.75)	U = 140.500; *p* = 0.493
CO, ppm, mean (SD)	21.33 (10.94)	19.17 (8.50)	U = 146.500; *p* = 0.623
Cotinine, mean (SD) ng/ml	2415.51 (1285.81)	3677.78 (3248.17)	U = 125.000; *p* = 0.502
QSU Factor 1, mean (SD)	3.16 (1.58)	3.26 (1.11)	U = 142.000; *p* = 0.527
QSU Factor 2, mean (SD)	1.84 (0.88)	1.93 (0.96)	U = 151.500; *p* = 0.739

CO, concentration of carbon monoxide (ppm) measured in expired air; FTND, Fagerström Test of Nicotine Dependence; QSU, Questionnaire on Smoking Urges; U, Mann–Whitney *U*

QSU Factor 1, “desire and intention to smoke and the anticipation of a positive outcome of smoking”; QSU Factor 2, “anticipation of immediate relief from nicotine withdrawal or relief from negative effect and the strong urge to smoke”

### Primary outcomes

#### Abstinence rate (smoking status nonsmoker)

**Fig. 2:**
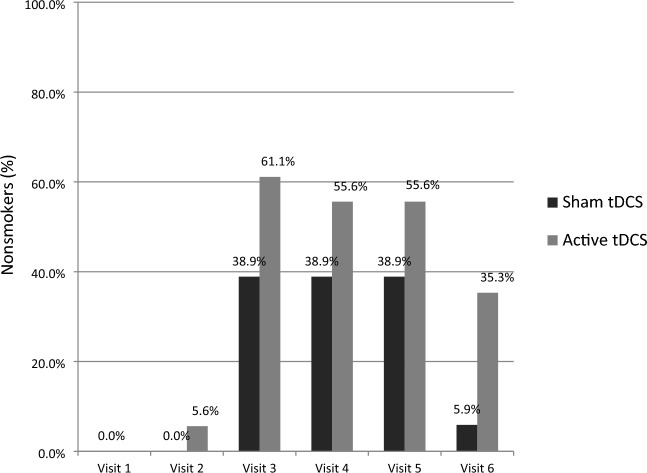
Number of nonsmokers in the groups receiving sham or active transcranial direct current stimulation followed by a brief intervention for smoking cessation. Visit 1 to 5 (transcranial direct current stimulation and brief intervention), n = 18 in each group; visit 6 (3-month follow-up), n = 17 in each group

At V1, none of the participants was a nonsmoker, but at V3, 7 of the 18 participants in the sham tDCS group and 11 of the 18 participants in the active tDCS group were nonsmokers. The numerical difference at V3 was not significant (*p* = 0.318). In the sham group, the number of nonsmokers remained unchanged until the end of treatment on day 5, but in the active group, 1 participant started smoking again. At follow-up, 1 of the 17 participants in the sham group was a nonsmoker and 6 of the 17 participants in the active group were, but the difference was not statistically significant (*p* = 0.085). The proportions of nonsmokers in the two groups at each study visit are shown in Fig. 2.

#### Urge to smoke assessed with the QSU

The results for Factor 1 of the QSU are shown in Table 4. The responses to the Factor 1 items showed no significant difference between the sham and active tDCS groups at any of the study visits, indicating that the level of desire and intention to smoke were the same in the two groups throughout the study. However, a longitudinal comparison of the data from the 6 study visits (with the Friedman test) showed a significant difference in the rank position of Factor 1 between the start and end of the study treatments, i.e., between V1 and V5 (Chi squared (5) = 18.027; *p* = 0.03; n = 34). Post hoc testing showed a greater difference between V1 and V5 in the active group than in the sham group (Dunn-Bonferroni test; z = 3.163, *p* = 0.023); the sham group showed no significant decrease of the QSU Factor 1 score from V1 to V5.

**Table 4:** Desire and intention to smoke and anticipation of a positive outcome of smoking assessed as Factor 1 on the Questionnaire on Smoking Urges

Study visit and day	QSU Factor 1 score, mean (SD)		Statistic and p value
	Sham tDCS (n = 18 except at V6, where n = 17)	Active tDCS (n = 18 except at V6, where n = 17)	
V1 on day 1	3.26 (1.11)	3.16 (1.58)	U = 142.000; *p* = 0.527
V2 on day 3	2.47 (0.92)	2.68 (1.22)	U = 155.000; *p* = 0.824
V3 on day 5	2.81 (1.44)	2.73 (1.48)	U = 152.000; *p* = 0.751
V4 on day 7	2.86 (1.58))	2.79 (1.61))	U = 155.000; *p* = 0.824
V5 on day 9	2.55 (1.44)	2.35 (1.43)	U = 143.000; *p* = 0.546
V6 at 3 months	2.88 (1.18)	2.93 (1.69)	U = 143.500; *p* = 0.972

V1–V5, transcranial direct current stimulation followed by brief intervention; V6, follow-up. QSU, Questionnaire on Smoking Urges; U, Mann–Whitney *U*; V1–V6: study visits 1 to 6

Group differences in Factor 2 of the QSU questionnaire were tested with the Mann–Whitney *U* test (Table 4). No significant difference was found in the Factor 2 score between the active and sham tDCS groups at any of the study visits, and a longitudinal comparison of the data showed no significant difference between the start and end of the study treatments (Table 5).

**Table 5:** Anticipation of immediate relief from nicotine withdrawal or relief from negative effect and the strong urge to smoke assessed as Factor 2 on the Questionnaire on Smoking Urges

Study visit and day	QSU Factor 2 score, mean (SD)		Statistic and *p* value
	Sham tDCS (n = 18 except at V6, where n = 17)	Active tDCS (n = 18 except at V6, where n = 17)	
V1 on day 1	1.93 (0.96)	1.84 (0.88)	U = 151.000; *p* = 0.739
V2 on day 3	1.92 (0.87)	1.93 (1.01)	U = 156.000; *p* = 0.849
V3 on day 5	2.15 (1.34)	2.00 (1.09)	U = 155.000; *p* = 0.824
V4 on day 7	2.30 (1.39)	1.84 (1.20)	U = 122.000; *p* = 0.201
V5 on day 9	1.99 (1.17)	1.63 (1.07)	U = 127.500; *p* = 0.248
V6 at month 3	1.99 (1.07)	1.54 (0.84)	U = 94.000: *p* = 0.079

V1–V5, transcranial direct current stimulation followed by brief intervention; V6, follow-up

QSU, Questionnaire on Smoking Urges; U, Mann–Whitney U; V1–V6: study visits 1 to 6

### Secondary outcomes

#### Number of cigarettes/day

**Fig. 3:**
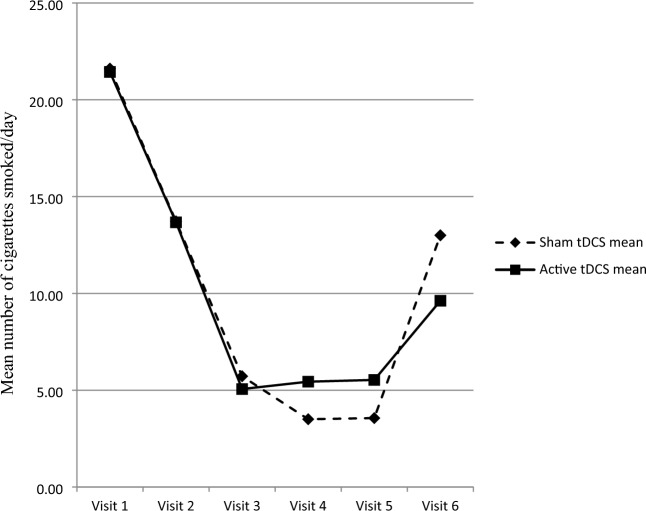
Number of cigarettes smoked per day in smokers receiving active or sham transcranial direct current stimulation followed by a brief intervention for smoking cessation. Visit 1 to 5 (transcranial direct current stimulation and brief intervention), n = 18 in each group; visit 6 (3-month follow-up), n = 17 in each group

The number of cigarettes smoked per day was not different between the active and sham tDCS groups at any of the 6 study visits (Mann–Whitney *U* test; V1, U = 140.50, *p* = 0.493; V2, U = 159.00, *p* = 0.924; V3, U = 134.50, *p* = 0.352; V4, U = 155.00, *p* = 0.815; V5, U = 152.00, *p* = 0.738; V6, U = 91.500, *p* = 0.066). Nevertheless, the low value at follow-up (V6) indicates that the number of cigarettes smoked per day was slightly lower in the active tDCS group than in the sham group. The mean scores at each study visit (V1 to V6) are shown in Fig. 3.

#### CO values in expired air

**Fig. 4:**
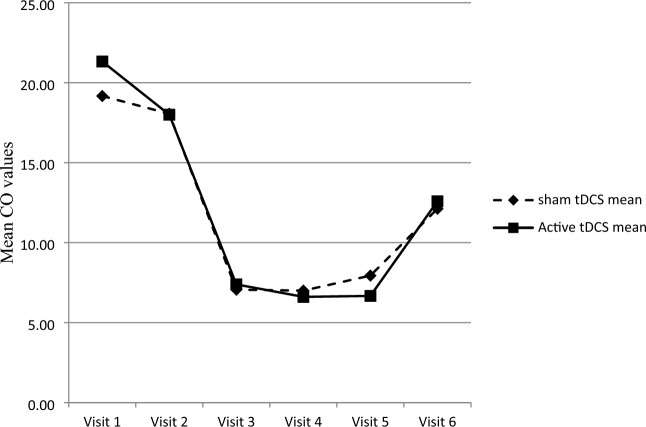
Carbon monoxide values in smokers receiving active and sham transcranial direct current stimulation followed by a brief intervention for smoking cessation. Visit 1 to 5 (transcranial direct current stimulation and brief intervention), n = 18 in each group; visit 6 (3-month follow-up), n = 17 in each group. CO, concentration of carbon monoxide (ppm) measured in expired air

No significant difference was found in the CO content of expired air at study visits V1 to V6 between the active and sham tDCS groups (Mann–Whitney *U* test; V1, U = 146.500, *p* = 0.623; V2, U = 156.500, *p* = 0.861; V3, U = 135.000, *p* = 0.366; V4, U = 152.000, *p* = 0.746; V5, U = 138,000, *p* = 0.430; V6, U = 138.500, *p* = 0.836). The mean values at study visits V1 to V6 are shown in Fig. 4.

#### Saliva cotinine values

We found no significant difference in cotinine values in saliva at study visits V1 to V5 between the active and sham tDCS groups (Mann–Whitney *U* test; V1, U = 125.000, *p* = 0.502; V2: U = 115.000, *p* = 0.637; V3, U = 96.000, *p* = 0.234; V4, U = 122.000, *p* = 0.614; V5, U = 115.000, *p* = 0.449). At study visit V6 (follow-up), the saliva samples were not analyzed because of organizational reasons.

**Fig. 5:**
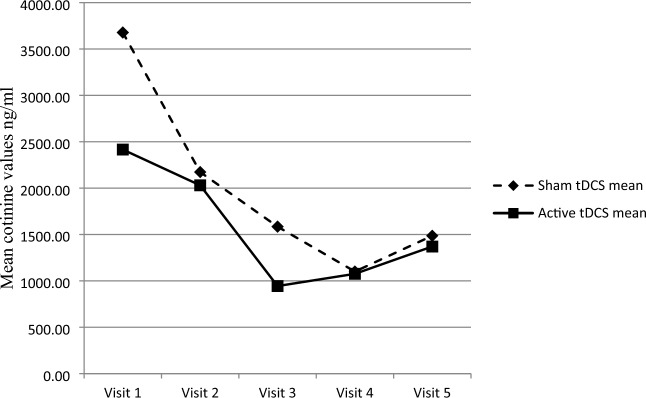
Cotinine values at study visits 1 to 5 in smokers receiving active and sham transcranial direct current stimulation followed by a brief intervention for smoking cessation. Visit 1 to 5 (transcranial direct current stimulation and brief intervention), n = 18 in each group; visit 6 (3-month follow-up), n = 17 in each group

The mean cotinine values are shown in Fig. 5.

#### Questionnaire on tobacco use

No significant difference in nicotine use in the past 3 months or 7 days before follow-up (V6) was found between the active and sham tDCS groups. The results are presented in Table 6.

**Table 6:** Assessment of nicotine use at follow-up

Questions*	Sham tDCS (N = 17)	Active tDCS (N = 17)	*p* values (Fisher’s exact test)
Question 1: Have you smoked since your last appointment?	Yes: 16 (94.1%) No: 1 (5.9%)	Yes: 12 (70.6%) No: 5 (29.4%)	*p* = 0.175
Question 2: Have you consumed nicotine in any other way since your last appointment?	Yes: 1 (5.9%) No:16 (94.1%)	Yes: 0 (0.0%) No: 17 (100%)	*p* = 0.100
Question 3: Have you smoked cigarettes in the last 7 days?	Yes: 16 (94.1%) No: 1 (5.9%)	Yes: 11 (64.7%) No: 6 (35.3%)	*p* = 0.085
Question 4: Have you consumed nicotine in any other way in the last 7 days?	Yes: 0 (0.0%) No: 17 (100%)	Yes: 0 (0.0%) No: 17 (100%)	
Question 5: If you smoked in the last 7 days, were there some days when you didn’t smoke a cigarette?	Yes: 5 (31.3%) No: 11 (68.7%)	Yes: 3 (27.3%) No: 8 (72.7%)	*p* = 1.000

*Participants could answer each question with *yes* or *no*

#### Questionnaire on side effects (CRQ)

Generally, side effects of stimulations were on a low level and active stimulations were well tolerated. Patients of both groups reported mild tingling and itching sensations, transient headache, and redness of the skin at the stimulation site. Side effects did not differ between active and sham stimulation, and no patient interrupted or quitted stimulation due to side effects.

## Discussion

The aim of this double-blind study was to evaluate the effects of tDCS of the DLPFC combined with a brief intervention on smoking cessation on the smoking cessation rate and urge to smoke. tDCS is a cost-effective, feasible noninvasive brain stimulation method that is described in the literature as a promising method for smoking cessation [7, 36].

The present study found no significant difference in smoking cessation rate between the sham and active tDCS groups, indicating that tDCS combined with a brief intervention for smoking cessation had no positive effect on the cessation rate. However, the data showed a slight trend towards positive effects of active tDCS in that the cessation rate was slightly higher than in the sham group, at least up to and including the fifth session, however this trend was not statistically significant. At the 3-month follow-up visit, the number of smokers had increased in both groups, so tDCS does not appear to have long-term effects when applied for a few stimulations. This finding agrees with a tDCS study by Fecteau et al. [37], which showed a decrease in the number of cigarettes smoked that lasted for only 4 days after the last tDCS session.

Similar to the studies by Boggio et al. [22] and Fregni et al. [3], the present study showed efficacy of tDCS in reducing the urge to smoke in that the QSU Factor 1 item *craving* improved significantly over the course of the 6 study visits in the active tDCS group compared with the sham group; no significant intergroup differences in QSU Factor 1 were found at any of the individual study visits. In contrast, the study by Xu et al. [38] used the same stimulation parameters and found no difference in the urge to smoke between active and sham tDCS; the study may not have found a difference because it included only 24 smokers and treated them with tDCS on only two days, which probably is insufficient to show group differences.

A strength of the current study was that it assessed the cessation rate and smoking status of participants objectively, i.e., by measuring the CO content of expired air or cotinine levels in saliva. In contrast, most studies on the efficacy of tDCS on nicotine addiction assess only the number of cigarettes smoked per day, which can lead to incorrect or biased findings because participants often cannot remember the exact number of cigarettes they smoked each day. However, the study also has some limitations. First, exclusion of concomitant mental disorders and/or substance use disorders was made by clinical diagnosis and was not underpinned by structured interviews. Second, unlike the study by Fecteau et al. [37], the tDCS sessions lasted 20 rather than 30 min; longer sessions may have led to significant differences between the active and sham groups. Third, tDCS was not performed on consecutive days. Last, the 10-min brief intervention after each tDCS session may have been too short to have a significant effect. Therefore, future studies should consider using longer tDCS sessions on consecutive days and a more intensive smoking cessation program. Larger sample sizes may also lead to statistically significant results.

## Conclusion

Although anodal tDCS was shown to improve craving for cigarettes in this study, abstinence rates did not differ between active and sham stimulation. To achieve sustainable effects and maintain abstinence, longer treatment periods or more intensive treatment programs (e.g., stimulation twice per day) probably are required. It also may be hypothesized that combination of anti-craving drugs, e.g., bupropion, with tDCS could strengthen prefrontal dopaminergic and noradrenergic transmission and therefore increase effects of concomitant psychotherapy by enhanced prefrontal cognitive control. Further studies are needed to address the combination of tDCS with a standardized psychotherapeutic intervention and/or anti-craving drugs in the treatment of addiction disorders.

## Appendix

### Follow-up questionnaire: Assessment of tobacco use

Name: _________________________________________

Date of birth: __________________________________

Date: ________________________________________

1. Have you smoked since your last appointment?

**Table:**

□ Yes	□ No

2. Have you consumed nicotine in any other way since your last appointment?

**Table:**

□ Yes	□ No

3. Have you smoked cigarettes in the last 7 days?

**Table:**

□ Yes	□ No

4. Have you consumed nicotine in any other way in the last 7 days?

**Table:**

□ Yes	□ No

If you smoked in the last 7 days, were there some days when you didn’t smoke a cigarette?

**Table:**

□ Yes	□ No

**If yes**, how many cigarette-free days were there? ______________ days

If you smoked in the last 7 days, how many cigarettes a day did you smoke on average on these "smoking days"?

________________ cigarettes/day

□ CO measurement: __________________ ppm

□ Saliva sample obtained
